# Culturally Tailored Cancer Communication, Education, and Research: The Highways and Back Roads of Appalachia

**Published:** 2009-03-15

**Authors:** Kelly A. Dorgan, Sadie P. Hutson, Katie L. Duvall, Gail Gerding

**Affiliations:** Department of Communication, East Tennessee State University; East Tennessee State University, Johnson City, Tennessee; East Tennessee State University, Johnson City, Tennessee; Boise State University, Boise, Idaho

## Introduction

We have varying experiences with Appalachia, yet we all agree that there is a unique relationship between Appalachians and cancer. Two of us are nurses who have worked with various communities. Two of us grew up here; 1 watched several of her relatives battle cancer in their Appalachian homes. All of us are scholars who want to talk with practitioners and researchers who are developing culturally tailored cancer control interventions. This goal to have a dialogue emerged after we had a series of discussions about cancer in Appalachia, discussions resulting in our developing a list of cultural traits that seem to be related to this region's high cancer morbidity and morality (Table). For example, in one of our previous publications we describe the association between the traditional Appalachian oral culture and the cancer experience, finding that cancer stories appeared to pass from 1 generation to the next (1). In turn, these stories seem to affect some community members' willingness to be screened. Our essay's purpose is not to justify the elements presented in the Table. Rather, we write to consider the following: What are the advantages and disadvantages of making generalizations about a culture that has already been marginalized by overgeneralizations?

We recognize that researchers and cancer control specialists need a road map of the cultures in which they work, a map highlighting values, norms, and beliefs. Such a cultural map is vital when updating cancer control plans and collaborating with Appalachian communities in cancer education efforts, for example. However, we have mixed feelings about defining a culture, in effect distilling Appalachians into manageable traits. Even for purposes of confronting the profound health disparities in this region (2), we are hesitant to publish a set of cultural characteristics that may be linked to Appalachia's high cancer mortality and morbidity.

In *Cultural Competence in Cancer Care: A Health Care Professional's Passport,* the Intercultural Cancer Council (ICC) provides a resource for practitioners working among other cultures, including rural Appalachia (3). Drawing on a passport metaphor, the authors attempt to introduce practitioners to other cultures "while not stimulating stereotypical thinking about the group" (3). Such well-intentioned writings — including our own — raise critical questions: 1) What is the value of summarizing a culture with a set of traits? 2) What are possible consequences of identifying cultural traits that may influence the cancer experience?

In this essay, we extend the ICC's use of the passport metaphor by journeying deeper into the topic of Appalachian cancer care. We seek to follow the highways that skirt this region and travel back roads to take a closer look at a diverse culture. Arguably, if there is still no agreement on where Appalachia begins and ends (4), then the debate about "what" Appalachia is will always be present. Although we may have good intentions in defining Appalachia for the purpose of designing cancer control campaigns and interventions, we should become comfortable with our ambivalence when it comes to reducing any culture to a compact list of traits, even when we do so for altruistic reasons. Ultimately, then, although we have to rely on cultural maps, we must remain ready to challenge the accuracy and helpfulness of such maps.

## Getting on the Road: Exploring Appalachia

This region, especially the more sparsely populated areas of Appalachia, is medically underserved and has disproportionately poor health compared with the rest of the nation, including higher rates of cancer and premature death (2,5). Documented challenges to providing better health care in this region include poor access to health insurance coverage and less contact with physicians (6). Driving through parts of Appalachia, the traveler may not be able to observe specific cultural characteristics and how they are linked to health disparities (7,8). What may be observable is how geographic isolation and economic conditions have combined to create unique health care challenges (9). Mountain roads may be narrower and provide less maneuverability than roads within a city, and they are traveled by heavy farm and timber-hauling vehicles, creating difficult driving conditions. Also complicating matters is that people may have to take time off from work, sometimes losing wages, to drive a long, winding road to a regional cancer center or the nearest community hospital.

Authors have argued that we must acknowledge the relationship between place and cancer (5). We know from our own work in communities that place matters when it comes to cancer control efforts. For example, 2 of us interviewed cancer control organizations working both inside and outside Appalachia. We were consistently told that interventions and programs targeted to Appalachian communities cannot be designed outside Appalachia. The reasons we were given: 1) visible and credible partnerships must be developed within the communities; 2) existing personal networks must be tapped into; and 3) cultural values, resources, and beliefs must be considered when engaging in cancer control in Appalachia. In this place, partnering with the community and using informal networks may be even more critical, especially if community members are suspicious of physicians and state agencies and prefer to get cancer information and advice from friends and family (1,10).

## Mapping Authentic Appalachia: A Necessary but Problematic Endeavor

In *Cultural Competence in Cancer Care*, the ICC provides a snapshot of rural Appalachians (3). This is an invaluable resource, but while consulting it, we must inquire: How do we best use such resources to help us map authentic Appalachia, especially because Appalachia is a place of diverse terrains, histories, and people? But in developing cultural maps, we should look beyond essays and handbooks. A source that helps us in our research is The University of Tennessee Press's nearly 2,000-page *Encyclopedia of Appalachia* (9). Of course, most practitioners and scholars, ourselves included, do not have the time to study such a book. So we have it on our bookshelves beside more compact references, such as the ICC handbook, allowing us to draw on a combination of quality resources.

For years, during our work with cancer-related projects, we have sought to understand and describe Appalachia. Like others, we have been exposed to stereotypes perpetuated about this region, sometimes by well-intended writers (11). Most of us deal with the reality that Appalachian communities are reticent when it comes to talking with journalists, researchers, and policy makers. When recruiting for new research projects on communication about cancer, we have consistently had to manage the legitimate concerns of many in this area: Will their participation in research help perpetuate the stereotypes of a feuding, impoverished, sullen, and fatalistic people that remain in our public narratives?

Over the years, we have worked diligently to prove our trustworthiness to Appalachian communities, communities often accustomed to being ignored, laughed at, or reduced to caricatures. In our research into how Appalachian women talk about and perceive the cervical cancer vaccine, the participants are simultaneously eager and resistant to participate. Only through our partnerships with credible community leaders and organizations have we been able to recruit diverse groups of women. Why have we encountered persistent and deep reluctance? Through discussions with community leaders and participants, we have discovered profound concerns that researchers, in their attempt to understand and describe Appalachia, will only perpetuate the "uneducated hillbilly" stereotype.

## Driving the Highways and Back Roads: Appalachia From a Distance and Up Close

In conducting research in Appalachia and with Appalachians, we find linguistic anthropologist Kenneth Pike's work helpful, specifically, his concepts of *etic* and *emic* (12). When studying human behavior at the cultural level, a researcher can use an etic perspective, which describes a culture from an external, outsider perspective. We liken the etic viewpoint to using interstates or highways: by taking this approach, drivers may form broad conclusions about distant communities. If the observer adheres to these broad conclusions too closely, this approach can result in overgeneralizations and harmful stereotypes. Conversely, those working from an emic perspective recognize that cultures comprise diverse subcultures, peoples, customs, and values. We liken this approach to a driver taking back roads through a community: a more intimate, immediate, and detailed map is created with closer observations of the differing communities within a community.

In our previous focus group work in Tennessee and Virginia about cancer and cancer communication in Appalachia, we found that broad cultural strokes (etic perspective) could help us in our cultural mapping. For example, commonalities existed regarding lack of access to health care services and an overarching lack of trust of researchers, health care practitioners, government, and agencies outside of Appalachia. We also collected diverse narratives across these focus groups regarding the experience with cancer in Appalachia. For example, participants in southwestern Virginia had familial and community identities shaped around the coal mining industry, which also shaped their perception of the cancer experience. Although the Virginia participants communicated pride about their mining heritage, they also conveyed concern about mining's possible link to the community's high incidences of cancer. The "mining narrative" was strikingly absent in the Tennessee focus groups. Anyone working in cancer control in Appalachia must pay attention to the broad cultural similarities and the within-culture differences to design effective educational programs and interventions.

## The Unwelcome Inhabitant: Cancer in Appalachia

In dealing with our ambivalence about defining the Appalachian culture, we have moved away from using terms such as "traits" or "characteristics." We prefer to use the term "signpost." Like the signs that appear along the road as we travel toward our destination, these signposts (Table) are not the destination itself. Rather, signposts are indicators pointing us toward a destination — a unique culture. For example, we are researching how the cervical cancer vaccine is perceived and talked about by Appalachian women. Some of our early findings suggest that the life priorities of Appalachian women may intersect with other signposts such as the role of religion to create a concern about being screened for and vaccinated against cervical cancer. These findings may help explain the high cervical cancer rates in Appalachia (5). We are also finding that tight family ties may intersect with self-reliance, making some Appalachian women rely on their personal networks for information about the vaccine rather than rely on their physicians.

In our ongoing research into cancer in Appalachia, we continue to consider these questions: 1) Are these signposts really suggesting a unique cancer experience in Appalachia? 2) What signposts are missing that may further explain the cancer experience in Appalachia? 3) How might these signposts be working together to create an experience with cancer in Appalachia that is different from cancer experiences in other parts of the United States? 4) How might the intersection of these signposts shift across diverse subcultures throughout north, central, and southern Appalachia? These questions will continue to guide our own research and our dialogues with other scholars and practitioners.

## Conclusion

Certainly, practitioners and researchers must strive to understand the cultural characteristics of various populations facing health disparities, including the African American, Latino and Hispanic, and Appalachian communities. However, we also know that in our search to understand and identify these characteristics, we may inadvertently perpetuate harmful stereotypes, all in an attempt to address disparities. Ultimately, then, we have decided to get comfortable with being uncomfortable when it comes to defining special populations, especially ones that have been traditionally ignored, painfully marginalized, and harmfully categorized. Like other cultures, Appalachia is — and will always be — beyond definition. Still, cancer continues to affect our Appalachian communities disproportionately, confronting generations with premature loss of family members while disrupting neighborhoods and communities.

Thus, our hands are forced: we must work with imperfect cultural maps, all the while being willing to dispose of those maps, including the map in the Table, when they no longer lead us to where we need to go. To fight cancer, we must journey deeper into Appalachia, not just pass by on the highways. We must be willing to take a turn onto that narrow mountain road or take a detour and drive straight into town where we will see the faces of everyday Appalachians. Those faces — our faces — reflect back to us the reasons we continue fighting cancer.

## Figures and Tables

**Table.** Signposts of the Cancer Experience in Authentic Appalachia

**Signposts**	**Examples**
Oral culture	Storytelling, singing, and preaching are important forms of communication. Family traditions and history tend to be handed down orally from one generation to another. Stories of cancer deaths predominate, leaving little hope of recovery.
Familism	Strong family bonds are present. Family members look to relatives for help and support in all aspects of life.
Self-reliance	A history of limited access to services, including health care, and strong antigovernment tendencies may have created a cultural norm emphasizing reliance on self, family, and community and the value of privacy.
Socially active rural people living within small geographically isolated communities	Established relationships with social groups (eg, family, church) may impede the formation of new associations (eg, volunteering for a local cancer coalition).
Competing life priorities	Health care, particularly preventive care, is often viewed as a luxury and is at the bottom of the family and personal priority list.
Role of religion	Religion is often the backbone of Appalachian residents. Churches (predominately Christian denominations) may be invaluable to people who put their health in the "hands of God."
People are rooted in mountains and the land	Family roots connecting rural Appalachians to the land tend to be deep and difficult to change or sever. To leave the mountains would cause separation from family, identity, and generational history.
Environmental influences	Some Appalachian residents have a love-hate relationship with industries that polluted water sources, contaminated air, or stripped land. Mountainous terrain actually helps hold in pollutants.
Cancer as a community disease	Cancer is seen as linked with both environmental causes and family history, affecting communities on multiple levels.
History of personal experience with cancer	In rural Appalachian communities, there is a sense that everyone is related to or knows someone who has suffered from cancer.
